# A Sensitive Two-Analyte Immunochromatographic Strip for Simultaneously Detecting Aflatoxin M1 and Chloramphenicol in Milk

**DOI:** 10.3390/toxins12100637

**Published:** 2020-10-02

**Authors:** Shih-Wei Wu, Jiunn-Liang Ko, Biing-Hui Liu, Feng-Yih Yu

**Affiliations:** 1 Graduate institute of Medicine, Chung Shan Medical University, Taichung 40201, Taiwan; shawn1024200288@gmail.com (S.-W.W.); jlko@csmu.edu.tw (J.-L.K.); 2 Graduate Institute of Toxicology, College of Medicine, National Taiwan University, Taipei 10051, Taiwan; 3 Department of Medical Research, Chung Shan Medical University Hospital, Taichung 40201, Taiwan; 4 Department of Biomedical Sciences, Chung Shan Medical University, Taichung 40201, Taiwan

**Keywords:** antibody, aflatoxin M1, chloramphenicol, ELISA, gold nanoparticle immunochromatographic strip

## Abstract

A two-analyte immunochromatographic strip (immunostrip) was developed for the simultaneous detection of aflatoxin M1 (AFM1) and chloramphenicol (CAP) in milk. Protein conjugates (AFM1-ovalbumin (OVA) and CAP-OVA) and goat anti-rabbit IgG were respectively drawn on nitrocellulose membrane as two test lines (T_1_ and T_2_) and a control line (C). The immunostrip was dipped into a well that contained a 200 μL milk sample, 5 μL AFM1 antibody-gold conjugates, and 8 μL CAP antibody-gold conjugates; the whole assay was completed in 15 min and the results could be interpreted visually or using a reader. This immunostrip has cut-off levels of 0.1 ng/mL and 0.5 ng/mL for AFM1 and CAP, respectively. Analysis of CAP and AFM1 in milk samples revealed that data from the immunostrip test agreed closely with those obtained from ELISA. The two-analyte immunostrip is a rapid way for on-site simultaneous detection of AFM1 and CAP in milk.

## 1. Introduction

Milk is consumed in daily life to provide essential nutrients for humans. Increasing concern about food safety has led to a growth in anxiety about the contamination of milk with mycotoxins or drug residues, such as aflatoxin M1 (AFM1) and chloramphenicol (CAP).

Aflatoxin M1, a hydroxylated metabolite of aflatoxin B1 (AFB1), is a frequently contaminant in dairy products since it remains stable during the whole process of milk pasteurization [1,2]. Previous studies have demonstrated that consumption of AFM1 through milk products is an important health issue to human, especially young individuals [3,4]. Aflatoxin M1 with similar adverse effects as AFB1 causes liver toxicity and reduces infant immunity and is also categorized as a human carcinogen (group 1) by the International Agency for Research on Cancer (IARC) [5]. Numerous countries have set legal regulations on AFM1 levels in milk products. The United States Food and Drug Administration (FDA) and the European Union (EU) restrict the concentrations of AFM1 in milk to 0.5 ng/mL and 0.05 ng/mL, respectively [6,7,8].

Chloramphenicol is a wide-spectrum antibiotic which has been commonly used as a veterinary drug since it was identified from *Streptomyces* species in 1947 [9]. Chloramphenicol is known to cause hematopoietic malfunction in human bone marrow and is also classified as probably carcinogenic to humans by the IARC [10,11]. Therefore, many nations, such as the USA, nations in the EU, Taiwan, and China, have set a limit for the usage of CAP in animal husbandry; the EU has regulated a maximum CAP residue level of 0.3 ng/g in milk [12].

Various analytical techniques, including ELISA and liquid chromatography, are widely used for the detection of AFM1 or CAP in milk because of the strict regulation on AFM1 and CAP worldwide [13,14,15,16,17]. With the advantage of accuracy, liquid chromatography has been used as a reference method for examining various small compounds. However, detecting low levels of AFM1 in milk with chromatographic methods generally needs a concentration and purification procedure through immunoaffinity columns, which is costly and time-consuming [18]. ELISA has also been applied in determining the levels of AFM1 and CAP residues for its speed, low-cost, and high-throughput ability. However, ELISA can only detect one target at a time and requires experimental equipment and professional handling.

The co-contamination of multi-toxic compounds in the food industry has urged the development of cost-effective and rapid methods for simultaneous detection of multi-analytes. The immunochromatographic strip is a simple, rapid, and multi-target technology suitable for on-site detection of natural toxins and drug residues by untrained personnel. Several studies demonstrate the use of immunostrip assays to determine the level of a single contaminant, either AFM1 or CAP, in milk samples [16,17,18,19,20]. In the present study, a two-analyte immunostrip assay was established for the first time to monitor AFM1 and CAP contamination at the same time with a concept of antigen competition. We have produced highly sensitive polyclonal antibodies against AFM1 or CAP and used them to develop direct competitive ELISAs (dcELISAs). The two-analyte immunostrip assay developed herein has low detection limits that can be used on-site to satisfy the relevant regulation of AFM1 and CAP in milk for all nations.

## 2. Results

### 2.1. Characterization of AFM1 and CAP Antibodies

**Figure 1:**
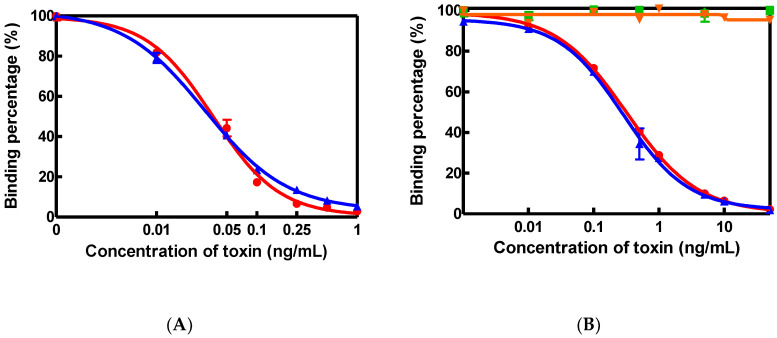
(**A**) Cross-reactivity of aflatoxin M1 (AFM1) polyclonal antibody with AFM1 (⚫) and aflatoxin B1 (AFB1) (▲) in a direct competitive ELISA (dcELISA). (**B**) Cross-reactivity of chloramphenicol (CAP) polyclonal antibody with CAP (⚫), CAP succinate sodium salt (CAP-SH) (▲), florfenicol (FF) (◼), and TAP (▼) in a dcELISA. All data were obtained based on the average of three sets of experiments. The absorbance of the control, A_0_, with no toxin present was 1.8.

Polyclonal antibodies specific to AFM1 or CAP were used to establish dcELISAs. In the AFM1-antibody based dcELISA, AFM1 at 0.02 ng/mL or AFB1 at 0.025 ng/mL were found to cause 50% inhibition (IC_50_) of AFM1-HRP binding to the AFM1 antibody, suggesting that the AFM1 antibody exhibited a high cross-reactivity with AFB1 (Figure 1A). The detection limit of AFM1 (IC_10_) in dcELISA was 0.002 ng/mL and the working scope IC_20_ to IC_80_ was 0.005 to 0.07 ng/mL. On the other hand, in the CAP dcELISA, the IC_50_ values of CAP and CAP succinate sodium salt (CAP-SH) for the binding of CAP-HRP to the CAP antibody was 0.21 and 0.27 ng/mL, respectively. The detection limit of CAP (IC_10_) was found to be 0.02 ng/mL, and the working scope IC_20_ to IC_80_ was 0.05 to 2.0 ng/mL (Figure 1B). The CAP antibody showed no cross-reactivity with florfenicol (FF) and thiamphenicol (TAP), two synthetic amphenicol antibiotics with similar structure and activity to CAP (Figure 1B).

### 2.2. Recovery Test of AFM1- or CAP-Spiked Milk Samples with dcELISA

Recovery tests were performed to investigate the accuracy of dcELISA in identification of AFM1 and CAP that were spiked in milk samples. As shown in Table S1, when the milk was spiked with AFM1 ranging from 0.05 to 5.0 ng/mL, the overall average recovery rate from the AFM1 dcELISA was 82.9% with a coefficient of variation (CV) value of 6.6. Additionally, the overall average recovery rate and CV value of the milk sample which had been spiked with 0.5 to 50 ng/mL CAP were found to be 80.7% and less than 3.9, respectively, in the CAP dcELISA (Table S2). These data suggest that both these two dcELISAs are feasible methods for detecting AFM1 and CAP in milk.

### 2.3. Determination of AFM1 and CAP Levels in Samples Using dcELISA 

The efficacy of dcELISA in measuring AFM1 or CAP concentration in milk samples was evaluated by gathering eleven milk and eight milk powder samples with trademark names from domestic stores. All the milk samples prepared in phosphate buffered saline (PBS)–bovine serum albumin (BSA)–Tween solution were subjected to AFM1 antibody-based or CAP antibody-based dcELISAs. The results of the dcELISAs indicated that all examined milk samples were AFM1-free (Table S3). Similarly, none of the milk sample extractions were found to be contaminated with the veterinary drug CAP (Table S3).

### 2.4. Construction of a Two-Analyte Immunostrip

The two-analyte immunostrip offers a simple and rapid method for on-site simultaneous testing of both AFM1 and CAP in milk samples. Both AFM1-OVA and CAP-OVA had been absorbed in the T_1_ and T_2_ test zones of the same membrane, respectively. When the AFM1 level in the milk sample exceeds a certain value, AFM1 toxins will occupy AFM1 antibody binding sites on all the AFM1 Ab–gold nanoparticles in the sample solution. Therefore, no free form of AFM1 Ab–gold nanoparticle is available to bind with the AFM1-OVA conjugate in the T_1_ test zone on the immunostrip membrane. Finally, no red signal from gold nanoparticles is visible in the T_1_ zone, indicating that the sample is AFM1-positive. Similarly, when the CAP level in the sample exceeds an established number, all the binding sites of CAP Ab–gold nanoparticles was saturated with CAP in the sample solution. There is no unbound Ab–gold particle able to bind with the CAP-OVA conjugate in the T_2_ test zone, so no red line in T2 zone suggests the milk sample is CAP-positive. 

**Figure 2:**
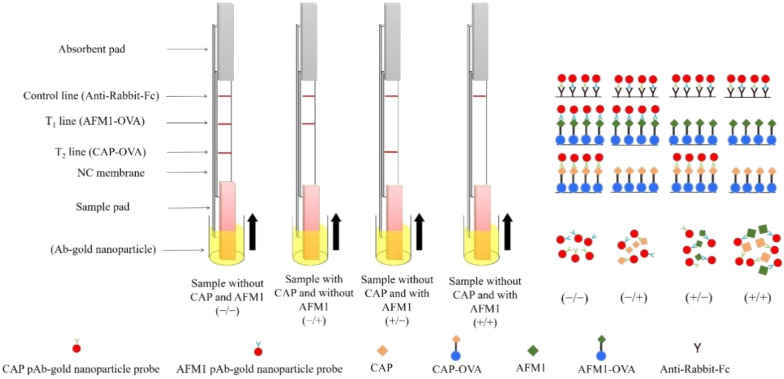
Schematic illustration of a two-analyte immunostrip. C, control zone (goat anti-rabbit IgG); T_1_, test zone (AFM1-OVA); T_2_, test zone (CAP-OVA). AFM1-OVA, CAP-OVA, and goat anti-rabbit IgG antibody were drawn onto two test lines and a control line of the nitrocellulose membrane, respectively. The CAP antibody (Ab)–gold nanoparticle conjugate, AFM1 polyclonal antibody–gold nanoparticle conjugate and milk sample were added to the sample well together. The assembling strip was dipped into the wells with the sample solution. After 15 min reaction, the test results were observed by eyes or measured by a scan reader. Arrow means move upward.

The control line on the immunostrip was absorbed with a goat anti-rabbit secondary antibody to verify the accuracy of testing procedure. In a properly conducted assay, a red line in the control zone should consistently appear, regardless of the presence of AFM1/CAP in the tested samples. Taken together, a milk sample free of both AFM1 and CAP should always yield three red lines (T_1_, T_2_, and control zones) on the membrane, whereas the AFM1 and CAP toxin-positive sample ought to only display one red line at control line (Figure 2).

### 2.5. Limit of Detection for AFM1 and CAP Immunostrip Assay

**Figure 3:**
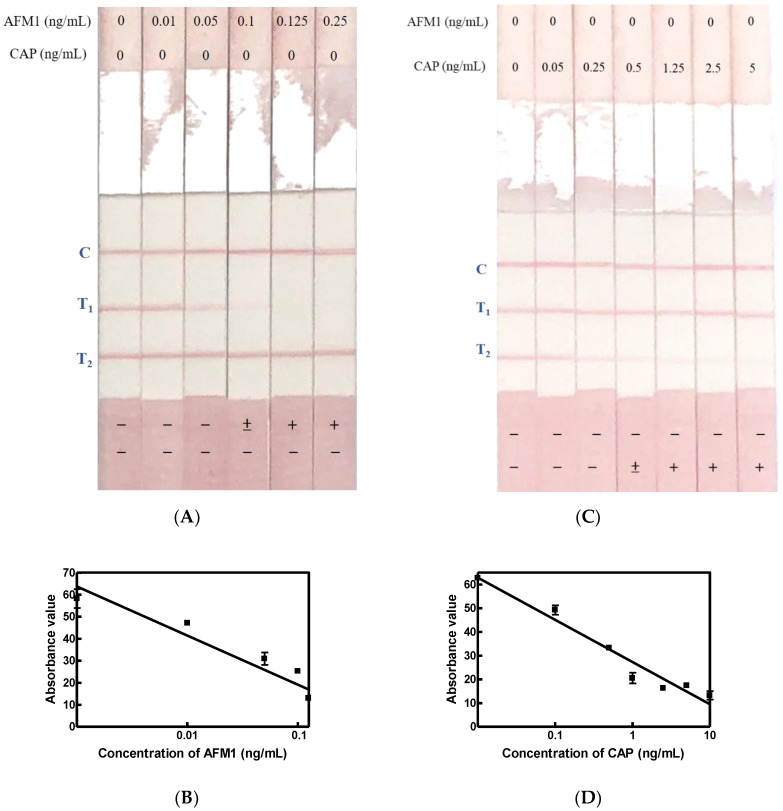
Detection limit of AFM1 and CAP with a two-analyte immunostrip. (**A**) A series of dilution (0–0.25 ng/mL) of certificated standard AFM1 was dissolved in PBS–BSA–Tween. Cutoff level is 0.1 ng/mL when the Ab–gold nanoparticle conjugates were loaded in the sample wells. (**B**) Standard curve of the T line color density value. When the scan reader value is less than 20, it indicates a positive result. Each concentration was tested for three repeats. (**C**) A series of dilution (0–5 ng/mL) of certificated standard CAP was dissolved in PBS–BSA–Tween. A CAP level exceeding 0.5 ng/mL resulted in the disappearance of the red line of T_2_, indicating that the sample is CAP positive. (**D**) If the scan reader value is less than 20, it indicates a positive result. C, Control line; T_1_, AFM1-OVA; T_2_, CAP-OVA.

Certified AFM1 standards at different concentrations (0, 0.01, 0.05, 0.1, 0.125, 0.25 ng/mL) were subjected to immunostrips on which AFM1-OVA and CAP-OVA had been absorbed in the T_1_ and T_2_ test zones. In Figure 3A, when both the AFM1 standard and the AFM1/CAP Ab–gold solution were mixed in wells of microplate at the same time, an immunostrip was subsequently inserted into the well for the capillary movement of the mixed solution. AFM1 at 0.1, 0.125, and 0.25 did not trigger any red signal in the T1 sites, indicating that the detection limit of AFM1 on the immunostrip is 0.1 ng/mL while the T line color density value was less than 30 (Figure 3B). Similarly, the application of certified CAP standard ranging from 0 to 5.0 ng/mL demonstrated that the CAP detection limit was 0.5 ng/mL, which was able to make the red line on T2 zone disappear (Figure 3C) and the color density of T line was less than 30 (Figure 3D).

### 2.6. Determination of AFM1 and CAP Levels in Spiked Samples Using Immunostrips

**Figure 4:**
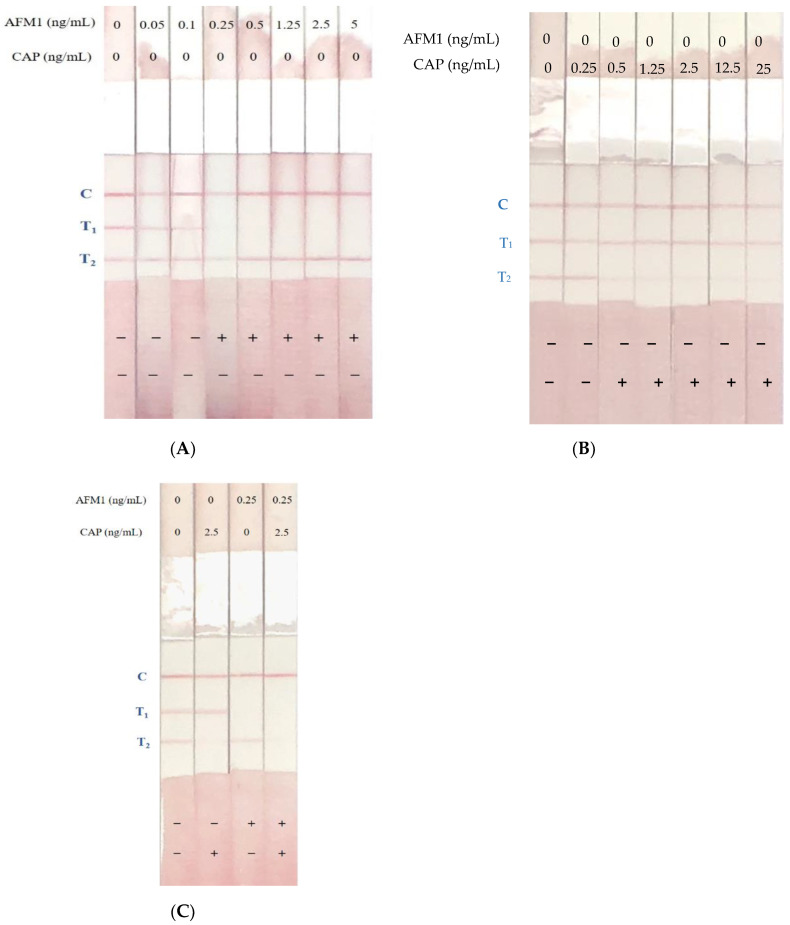
Detection of spiked AFM1 or CAP milk samples with immunostrips. (**A**) The spiked AFM1 (0–5 ng/mL) milk samples were subjected to the immunostrip assay. The cutoff level was 0.25 ng/mL when antibody–gold nanoparticle conjugates were loaded in the sample wells. (**B**) The spiked CAP (0–25 ng/mL) milk samples were subjected to the immunostrip assay. The cutoff level was 0.5 ng/mL when Ab–gold nanoparticle conjugates were loaded in the sample wells. (**C**) The spiked milk sample with either AFM1 levels at 0.25 ng/mL or CAP levels at 2.5 ng/mL caused the T_1_ or T_2_ lines’ disappearance.

To confirm the effectiveness of immunostrips for analyzing contaminated samples, the extraction solution of milk samples with final spiked concentrations of AFM1 and CAP from 0–5 ng/mL and 0–25 ng/mL, respectively, were subjected to immunostrip assays. Figure 4A presents the results of the AFM1-spiked samples. When the milk samples with AFM1 levels reached 0.1 ng/mL, the red line at the T_1_ zone looked blurry; the color of the T_1_ line completely faded away at 0.25 ng/mL AFM1. On the other hand, for those milk samples with CAP levels above 0.5 ng/mL the red line at T_2_ zone was able to disappear, which is corresponding to the application of CAP standards (Figure 4B). Milk samples with both AFM1 levels at 0.25 ng/mL and CAP levels at 2.5 ng/mL caused the T_1_ and T_2_ lines to disappear (Figure 4C).

### 2.7. Results of Naturally Contaminated Samples

**Figure 5:**
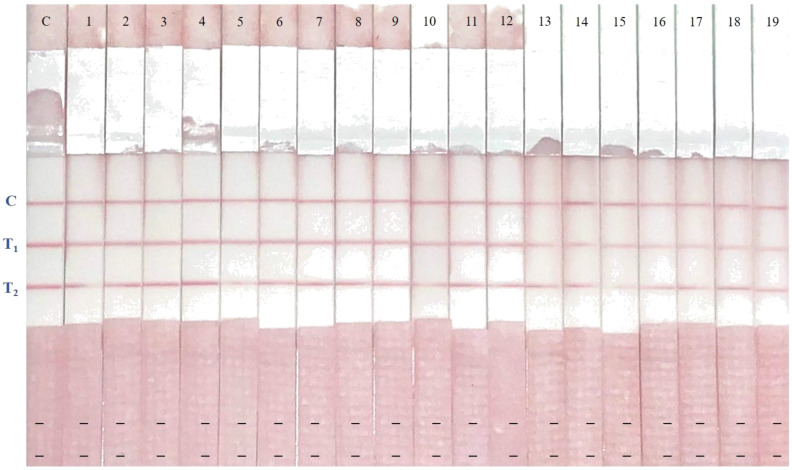
Analysis of milk samples with two-analyte immunostrip. Detection of AFM1 and CAP with immunostrips in 11 milk samples, eight milk powder samples, and one control sample, with two-analyte immunostrips. All samples showed three red lines (T_1_, T_2,_ and C) on the membrane indicating that they are free of AFM1 and CAP. All of the milk samples containing AFM1 lower than 0.1 ng/mL showed a red line (T_1_) on the membrane, indicating that they are AFM1 negative. All of the milk samples containing CAP lower than 0.5 ng/mL showed a red line (T_2_) on the membrane, indicating that they are CAP negative. C, control line; T_1_, AFM1-OVA; T_2_, CAP-OVA.

A control, 11 milk samples, and eight milk powder samples were applied to a well to test either AFM1 or CAP contamination with immunostrips. Table S3 shows that all tested samples were negative for both AFM1 and CAP in the dcELISA and the immunostrips also yielded negative results for AFM1 and CAP in all milk samples with two clear red lines (T_1_ and T_2_) and another red line in the control zone (Figure 5).

## 3. Discussion and Conclusions

The mycotoxin AFM1 and the antibiotic CAP have very different chemical structures. They may be present in milk and are hazardous to humans when consumed. Antibodies specific to either AFM1 or CAP were generated in our laboratory. Using these antibodies, rapid dcELISAs were established for AFM1 and CAP. The developed dcELISA for AFM1 had a detection limit of 0.002 ng/mL and IC_50_ of 0.02 ng/mL. The dcELISA sensitivity obviously preceded that reported by Pei et al. (2009) [21] who achieved a detection limit of 0.04 ng/mL and an IC_50_ of 0.62 ng/mL. In contrast, the dcELISA for CAP herein had a detection limit of 0.01 ng/mL and an IC_50_ of 0.15 ng/mL. The dcELISA sensitivity is superior to that the previous article, which had an IC_50_ value of 0.3 ng/mL [22]. Analytical recoveries of AFM1- or CAP-spiked milk samples using dcELISA were performed. The analytical recovery rates for AFM1 was in the range 74.3–81.0% with coefficients of variation of less than 10%. The analytical recovery rates for CAP were in the range 71.6–90.1% with coefficients of variation of 0.4–3.9%. These data suggest that the two dcELISAs can be applied to detect AFM1 and CAP.

In this work, a two-analyte immunostrip was established to detect the contamination of milk samples with AFM1 and CAP. The results of this immunostrip are visible in 15 min. Therefore, simultaneous detection of AFM1 and CAP contaminants in milk becomes conveniently and quickly possible. On the two-analyte immunostrips, the different coating antigens for AFM1-OVA and CAP-OVA were immobilized onto the T1 and T2 test lines of the strips, respectively. Figure 2 presents the explicitness of AFM1 Ab–gold and CAP Ab–gold at the correlated test lines. The major purpose of immunostrip assay was on-site simultaneous qualitative testing of AFM1 and CAP contaminants in milk at regulatory level. In our previous study, we developed an AFM1 immunostrip with a detection limit of 0.5–1 ng/mL [23]. An AFM1 immunostrip developed by Zhang et al., (2013) [20] had a detection limit of 0.2 ng/mL. In their assay system, they used a sample solution premixed with antibody–colloidal gold conjugates prior to the test without using the sample pad. This study adapted premixing of the sample and Ab–gold nanoparticle conjugates to yield a detection limit of 0.1 ng/mL. This level is below the regulatory limit for AFM1 in milk in the United States. Our work focuses on the development of a sensitive immunostrip, and several parameters, such as the pH value of the gold nanoparticle solution for conjugation, the quantity of antibody for gold nanoparticle conjugation, antigen-protein reagents, and nitrocellulose (NC) membrane, were optimized. In this study, both AFM1 Ab–gold and CAP Ab–gold were coupled in a borax buffer (pH 6.0) to maximize the sensitivity of the immunostrip test. The optimal amounts of anti-AFM1 antibody and anti-CAP antibody were about 7 μg and 18 μg, respectively, for 2 mL gold nanoparticles. The previous works [24,25] developed a fluorescent-based immunostrip to detect AFM1 or CAP in milk. Their AFM1 and CAP detection limits of their immunostrips were below 0.1 ng/mL, but extra equipment was required to obtain the fluorescent signal, making them impossible to use by the general public. Additionally, those immunostrips did not detect AFM1 and CAP at the same time.

The previous study found that the signal at the test line close to the sample pad was stronger than that farther from the sample pad [26]. However, in this work, the test line close to the sample pad was less sensitive than that which was away from the sample pad. To compare the positions of AFM1-OVA and CAP-OVA in the test line, the AFM1-OVA and CAP-OVA were drawn into the T_1_ zone and T_2_ zone, respectively. CAP-OVA and AFM1-OVA were also drawn into the T_1_ zone and T_2_ zone, respectively. When AFM1-OVA and CAP-OVA were drawn into the T_1_ and T_2_ zones, respectively, the immunostrip had the highest sensitivity and yielded the strongest signal (data not shown). The detection limit of the immunostrip is interpreted as the levels of AFM1 and CAP that lead to complete disappearance of the T_1_ and T_2_ lines. To confirm the detection limit of the immunostrip test, consecutive AFM1 and CAP standard solutions were tested five times using the immunostrip as described above. The resulting color line densities were measured using a strip scan reader. Figure 4 presents the sensitivity of the immunostrip assay for various levels of AFM1 and CAP. Due to the interaction between the spiked AFM1 and CAP in milk samples, the levels of AFM1 reached to 0.25 ng/mL and CAP levels reached 2.5 ng/mL which caused the T_1_ and T_2_ lines’ disappearance (Figure 4C). The previous report mentioned that milk samples contain many sugars, fats, and protein. These matrices may affect the binding of antigens and antibodies, leading to false-positive results [17]. Therefore, we used PBS–BSA–Tween to dilute the milk samples before testing to prevent matrix interference. However, a relatively high concentration of BSA (0.5%) may weaken the signal of the immunostrip and a lower concentration of BSA (0.1%) may cause high matrix interference. Hence, the effects of various concentrations of BSA (0.1–0.5%) were compared. The results revealed that 0.5% was the best concentration of BSA to prevent matrix interference and yield an acceptable signal on the immunostrip (data not shown).

This work developed a two-analyte immunostrip for the simultaneous detection of AFM1 and CAP in milk. It is the first time that an immunostrip that can simultaneously identify AFM1 and CAP in milk has been created. This immunostrip test could be completed in 15 min and has cut-off levels of 0.1 ng/mL and 0.5 ng/mL for AFM1 and CAP, respectively. The results demonstrate that this two-analyte immunostrip test is a rapid and reliable tool for the on-site simultaneous detection of AFM1 and CAP in milk.

## 4. Materials and Methods

Aflatoxin M1 (AFM1), AFB1, chloramphenicol (CAP), CAP succinate sodium salt (CAP-SH), florfenicol (FF), hemocyanin keyhole limpet (KLH), carboxymethoxylanmine hemihydrochloride (CMO), 1-ethyl-3-[3-dimethylaminopropyl]-carbodimide (EDC), N-hydroxysuccinimide (NHS), N,N-dicyclohexylohexylcarbodiimide (DCC), sodium bicarbonate, ovalbumin (OVA), bovine serum albumin (BSA), hydrogen tetrachloroaurate, and dimethyl sulfoxide (DMSO) were purchased from Sigma (St. Louis, MO, USA). Horseradish peroxidase (HRP) was obtained from Roche (Mannheim, Germany). K-Blue TMB substrate (3,3′, 5,5′-tetramethylbenzidine) was brought from Neogen Corp (Lexington, KY, USA). Sodium carbonate, Tween 20, and ammonium sulfate were obtained from Merck (Darmstadt, Germany). Freund’s complete/incomplete adjuvants, goat anti-rabbit IgG, and microtiter plates were obtained from Thermo (East Grinstead, UK). All strip components were obtained from MDI Technologies (Ambala, India). The Troy Double Axes Programmable Controller (Taichung, Taiwan) was used to draw the test and the control line on the immunostrip. An ELISA reader (Vmax) was from Molecular Devices Co. (Menlo Park, CA, USA). A strip scan reader was purchased from Taiwan Advance Bio-Pharmaceutical Inc. (Taipei, Taiwan).

### 4.1. Preparation of Various AFM1 Conjugates and CAP Conjugates

#### 4.1.1. Conjugation of AFM1 to OVA or HRP

The conjugation protocol of AFM1-CMO was carried out as previously described [20] and then the AFM1-CMO was conjugated to OVA or HRP as earlier described [15]. AFM1-CMO-OVA (also named AFM1-OVA) and AFM1-CMO-HRP (also named AFM1-HRP) were used for the test lines of the immunostrip and dcELISA, respectively.

#### 4.1.2. Conjugation of CAP-SH to OVA or HRP

The CAP-SH was conjugated to either OVA or HRP as previously described and then used as an antigen for test line 2 on the immunostrip [17]. Briefly, CAP-SH (1 mg in 0.1 mL DMSO), EDC (2 mg in 0.02 mL DMSO), and NHS (1.5 mg in 0.015 mL DMSO) were mixed together with constant stirring at room temperature for 2 h. Next, 16 mg OVA (1 mL 0.01 M PBS, pH 7.6) was added to the reactant with gentle stirring overnight at 25 °C. The reactant was dialyzed in 0.01 M PBS for 48 h and then stored at −18 °C until used. CAP-SH-OVA and CAP-SH-HRP were used for test line 2 the of immunostrip and dcELISA, respectively.

### 4.2. Generation of Polyclonal Antibody

Both polyclonal antibodies against AFM1 or CAP were generated according to previous reports [17,23]. These antibodies were used to conjugate with gold nanoparticles for the immunostrips or as coating antibodies for dcELISA.

### 4.3. dcELISA

The dcELISA was conducted as previously described, with a slight modification [27,28]. The polyclonal antibodies specific to AFM1 or CAP were coated onto a 96-well plate (100 μL of 2 μg/mL in 0.01 M PBS) and incubated at 37 °C for 1 h. After being washed with PBS–Tween (0.05% Tween 20 in 0.01 M PBS, 0.35 mL per well), the plates were blocked with 170 μL BSA–PBS (0.1% BSA in PBS) at 37 °C for 30 min before being washed with a PBS–Tween solution. The AFM1 or CAP standard (0.05 mL/well), ranging from 0 to 100 ng/mL, or sample extracts were added into ELISA plates together with the AFB1-CMO-HRP or CAP-SH-HRP conjugate (50 μL/well) and then reacted at 37 °C for 1 h. The plate was washed with PBS–Tween again and 0.1 mL of TMB substrate was added to each well for color development at room temperature for 20 min before addition of 0.1 mL 1 N HCl to stop the reaction. A Vmax automatic ELISA reader was used to determine the absorbance at 450 nm.

### 4.4. Milk Sample Preparation

For the spiked assay to ensure the detecting accuracy of the immunostrips, the liquid milk samples, collected from a local store and analyzed to be AFM1 and CAP negative with dcELISA, were spiked with AFM1 ranging from 0.05 to 0.5 ng/mL or with CAP ranging from 0.5 to 50 ng/mL. On the other hand, eleven liquid milk samples and eight milk powder samples were obtained from community stores in order to examine both levels of AFM1 and CAP. Ten milliliters of each liquid milk sample was centrifuged at 18,500× *g* for 15 min at 4 °C. Subsequently, the middle layer was collected and diluted with the same volume of PBS–BSA–Tween solution (0.5% BSA and 0.05% Tween 20 in 0.01 M PBS) for the following experiments. To analyze the AFM1 and CAP levels in the milk powder samples, five grams of each milk powder was extracted in 10 mL PBS–BSA–Tween solution and shaken at room temperature for 30 min. The mixture was centrifuged at 18,500× *g* for 15 min at 4 °C and the supernatant was collected for further immunostrip and dcELISA analysis.

### 4.5. Coupling the Antibody-Gold Nanoparticle (Ab-Gold)

Gold nanoparticles were first synthesized according to the previous report [17]. To further adsorb antibodies and serve as probes in immunostrips, 7 μg of AFM1 or 18 μg of CAP antibody in 0.1 mL of borax buffer (pH 6.0) were added dropwise to 2 mL of gold nanoparticle solution with constant stirring at room temperature for 1 h. Subsequently, 200 μL of BSA solution was added to stop the reaction for 30 min, centrifuged at 13,000× *g* rpm for 30 min at 4 °C, and then the supernatant was discarded. The pellets containing AFM1 Ab–gold or CAP Ab–gold nanoparticles were reconstituted in 0.2 mL of 20 mM Tris-buffer saline and stored at 4 °C for further usage [29].

### 4.6. Immunostrip Assembly

The components of the immunostrip consisted of a sample pad, an absorbent pad, and an NC membrane with a control zone and test zone, as shown in Figure 2. The control and test zones on the NC membrane were absorbed with 0.25 μL of AFB1-OVA conjugate (5 mg/mL), 0.25 μL of CAP-OVA conjugate (3 mg/mL), and 0.25 μL of goat anti-rabbit secondary antibody (2 mg/mL) on T_1_, T_2_, and control areas, respectively. After air drying for 10 min at room temperature, the immunostrip was constructed according to the report of Liu et al. (2008) [30]

### 4.7. Determining AFM1 and CAP Levels in Milk Samples with Immunostrip

The AFM1 Ab–gold (8 μL) and CAP Ab–gold (5 μL) were combined with a milk sample solution (200 μL), AFM1 standard solution or CAP standard solution. Each of the sample solutions, the various level of AFM1 standard solutions (0–0.25 ng/mL), and CAP standard solutions (0–10 ng/mL) were added to the sample well. The immunostrips were put into the sample wells and the milk samples and AFM1 or CAP standard solutions moved along the membrane by capillary action. The visual results on the immunostrip were ascertained after 15 min and a strip scan reader was used to read the red line color density.

### 4.8. Data Analysis

Sample and standard in the dcELISA were analyzed in triplicate, and the average value was obtained. GraphPad prism 5.0 software fitted to a four-parameter logistic equation was applied to a standard curve by plotting the absorbance value against the logarithm of the analyte concentrations:

Y = {(A − D) ÷ (1 + (x/C)^B^)} + D

where A is the absorbance without an analyte (A_max_), B is the curve slope inflection point, C is the x value at the inflection point, and D is the background signal (A_min_).

## Supplementary Materials

The following are available online at https://www.mdpi.com/2072-6651/12/10/637/s1, Table S1: Recovery of Aflatoxin M1 from spiked milk samples. Table S2. Recovery of Chloramphenicol from spiked milk samples. Table S3. ELISA and immunostrip analysis of AFM1 and CAP in milk samples.
