# Supplementary material for: A Sensitive Two-Analyte Immunochromatographic Strip for Simultaneously Detecting Aflatoxin M1 and Chloramphenicol in Milk
Source: Toxins (Basel). 2020 Oct 2;12(10):637. doi: 10.3390/toxins12100637 (PMC7600427; doi:10.3390/toxins12100637)
Supplement: Supplementary file 1 [file toxins-12-00637-s001.pdf]

Supplementary Materials: A Sensitive Two-Analyte Immunochromatographic Strip for Simultaneously Detecting Aflatoxin M1 and Chloramphenicol in Milk

Shih-Wei Wu, Jiunn-Liang Ko, Biing-Hui Liu and Feng-Yih Yu

Table S1. Recovery of Aflatoxin M1 from spiked milk samples.

Spiked AFM1 (ng/mL)	ELISA (ng/mL)	CV (%)	Recovery (%)
0.05	0.04 ± 0.002	0.7	80.0
0.1	0.09 ± 0.003	4.5	90.0
0.25	0.21± 0.002	9.5	84.0
0.5	0.44 ± 0.05	9.1	88.0
1.25	1.07 ± 0.04	2.9	85.6
2.5	1.96 ± 0.06	3.1	78.4
5	3.72 ± 0.30	8.1	74.4
Overall mean		6.6	82.9

Table S2. Recovery of Chloramphenicol from spiked milk samples.

Spiked CAP (ng/mL)	ELISA (ng/mL)	CV (%)	Recovery (%)
0.5	0.36 ± 0.016	3	72.0
1.0	0.84 ± 0.02	2.7	84.0
2.5	2.16 ± 0.06	3.9	86.4
5.0	3.81 ± 0.03	0.7	76.2
25	18.87 ± 0.01	0.4	75.5
50	45.03 ± 0.01	0.4	90.1
Overall mean		1.4	80.7

Table S3. ELISA and immunostrip analysis of AFM1 and CAP in milk samples.

Samples	Food	ELISA (ng/mL)[a]	ELISA (ng/g)[a]	Immunostrip Assays
1	Milk	ND		−,−
2	Milk	ND		−,−
3	Milk	ND		−,−
4	Milk	ND		−,−
5	Milk	ND		−,−
6	Milk	ND		−,−
7	Milk	ND		−,−
8	Milk	ND		−,−
9	Milk	ND		−,−
10	Milk	ND		−,−
11	Milk	ND		−,−
12	Milk Powder	ND	ND	−,−
13	Milk Powder	ND	ND	−,−
14	Milk Powder	ND	ND	−,−
15	Milk Powder	ND	ND	−,−
16	Milk Powder	ND	ND	−,−
17	Milk Powder	ND	ND	−,−

18	Milk Powder	ND	ND	–,–
19	Milk Powder	ND	ND	–,–

[a] Each sample was extracted duplicate and each extract was analyzed in duplicate. ND, not detected.
